# New Baitouweng Decoction alleviated DSS-induced colitis through the FXR/NLRP3 signaling pathway by regulating gut microbiota and bile acids

**DOI:** 10.1093/gastro/goaf055

**Published:** 2025-06-26

**Authors:** Li Liu, Zhi-Wei Miao, Yu-Zhuo Wei, Shu Bu, Xin Gu, Yi Xu, Zhao-Wei Shan

**Affiliations:** Nanjing University of Chinese Medicine, Nanjing, Jiangsu, P. R. China; Gastroenterology Department, Affiliated Hospital of Nanjing University of Chinese Medicine, Nanjing, Jiangsu, P. R. China; Gastroenterology Department, Affiliated Hospital of Nanjing University of Chinese Medicine, Zhangjiagang, Jiangsu, P. R. China; Nanjing University of Chinese Medicine, Nanjing, Jiangsu, P. R. China; Gastroenterology Department, Affiliated Hospital of Nanjing University of Chinese Medicine, Nanjing, Jiangsu, P. R. China; Nanjing University of Chinese Medicine, Nanjing, Jiangsu, P. R. China; Gastroenterology Department, Affiliated Hospital of Nanjing University of Chinese Medicine, Nanjing, Jiangsu, P. R. China; Nanjing University of Chinese Medicine, Nanjing, Jiangsu, P. R. China; Gastroenterology Department, Affiliated Hospital of Nanjing University of Chinese Medicine, Nanjing, Jiangsu, P. R. China; Gastroenterology Department, Affiliated Hospital of Nanjing University of Chinese Medicine, Nanjing, Jiangsu, P. R. China; Nanjing University of Chinese Medicine, Nanjing, Jiangsu, P. R. China

**Keywords:** New Baitouweng Decoction, ulcerative colitis, gut microbiota, bile acids, FXR–NLRP3 pathway

## Abstract

**Background:**

Ulcerative colitis (UC) is a chronic disease that induces colon tissue damage. Previous studies have shown the clinical benefit of New Baitouweng Decoction (NBD). Here, we aimed to investigate the effects of NBD on dextran sodium sulfate (DSS)-induced UC and the underlying mechanisms in a mouse model.

**Methods:**

UC was induced in mice by using DSS for 7 days. The efficacy of NBD was determined by analysing the pathological appearance and the expression of inflammatory factors and tight junction proteins. 16S rDNA sequencing was used to describe the gut microbiota. Gas chromatography–mass spectrometry was employed to quantify bile acid (BA) levels. Spearman’s correlation analysis was conducted to determine the relationship between gut microbiota composition and BA profiles. Western blot was used to detect the amounts of farnesoid X receptor (FXR), Nod-like receptor (NLR) family pyrin domain containing 3 (NLRP3), caspase-1, and cleaved caspase-1.

**Results:**

NBD reduced the disease activity index scores, ameliorated colonic pathological damage, inhibited colon inflammation, and repaired the intestinal barrier. In addition, 16S rDNA sequencing showed that NBD enhanced the relative abundance of beneficial bacteria such as *Lactobacillus* and *Akkermansia*, known to be involved in fecal BA metabolism. Furthermore, BA metabolomics analysis indicated that NBD elevated the concentrations of lithocholic acid and deoxycholic acid, thereby linking to the activation of the FXR pathway to inhibit NLRP3-mediated inflammation. Inhibiting FXR activation by using Z-guggulsterone impeded the protective function of NBD in DSS-induced UC.

**Conclusion:**

NBD had a therapeutic effect on DSS-induced UC in a mouse model by regulating the gut microbiota, BAs, and subsequent FXR–NLRP3 pathway for decreasing the release of pro-inflammatory factors and repairing the intestinal barrier to preserve the equilibrium.

## Introduction

Ulcerative colitis (UC)—a very common chronic disease—is characterized by a disorder of the inflammatory process and damage to the colon tissues [1]. The mechanism of the occurrence of UC is complex and unclear, but it is well accepted that it is related to various factors such as genetics factors, the external environment, autoimmune dysfunction, and intestinal bacterial imbalance [2]. Recently, the drugs for UC, although effective, have shown gastrointestinal side effects, hepatotoxicity, tolerance, and high conventional cost [3]. Hence, there is an urgent need to find new therapeutic medications for the treatment of UC.

There has been increasing evidence suggesting a close relationship between gut microbes and bile acids (BAs), resulting in the proposal of the” gut microbiota–bile acid axis” [4]. Recent research has shown that gut microbiota imbalances are an important factor in the occurrence and development of UC. Gut bacterial balances alter microbial diversity, thereby destroying the symbiosis between microbials and hosts [5]. Gut microbiota disorders increase the amounts of harmful bacteria, causing intestinal stagnation [6]. In addition, intestinal microbiota-derived metabolites can trigger the colon mucosal immune response, thus destroying the intestinal mucous membrane immune function and causing UC [7]. BA is a metabolite associated with the microbiome that regulates intestinal homeostasis and inflammation in the UC [8]. Some BAs, such as deoxycholic acid (DCA) and lithocholic acid (LCA), have been individually confirmed to possess cytotoxicity to hepatic cells and cause colonic inflammation [9]. Thus, maintaining the balance of the microbiota and BA metabolite system could be a promising strategy for the treatment of UC. In addition, BAs can regulate enterohepatic circulation by activating the farnesoid X receptor (FXR) [10]. In UC, FXR activation suppresses inflammation of the intestine and intestinal barrier [11]. Recent convincing research has demonstrated that FXR negatively regulates the activation of Nod-like receptor pyrin domain containing 3 (NLRP3) [12], which plays a crucial role in regulating gut homeostasis, including controlling the integrity of the upper intestine and regulating the intestinal immune response [13]. Hence, regulating the gut microbiota–BA disorder could suppress inflammation by activating FXR and inhibiting the NLRP3 signaling pathway in UC.

Given the less-poisonous side effects and flexibility, traditional Chinese medicine (TCM) has become a common alternative treatment for UC. Baitouweng decoction, originating from Zhang Zhongjing’s”Shanghan Theory,” is a classic Chinese medicine prescription. Several studies of Baitouweng decoction have shown the functions in anti-diarrhea, anti-inflammation, and gut microbiome regulation [14]. Based on clinical experience, in order to better treat UC, several herbs were further added to the Baitouweng decoction to form the New Baitouweng Decoction (NBD). The effectiveness of NBD towards UC treatment had been demonstrated in our previous clinical studies [15]. Furthermore, our earlier studies showed that the transplantation of NBD joint fecal flora could alleviate dextran sodium sulfate (DSS)-induced UC in rats by regulating gut microbiota metabolic homeostasis and the STAT3/NF-κB signaling pathway [16]. However, more work is needed to explore the efficacy and mechanisms of NBD in the amelioration of DSS-induced UC. Therefore, we hypothesized that NBD could alleviate UC by regulating the intestinal flora and BAs. We firstly established the DSS-induced UC model in C57BL/6 mice. Using this model, we determined the therapeutic effect of NBD on UC through detecting the disease activity index (DAI) scores, inflammatory factors, Occludin, and ZO-1 proteins of colon tissues; the morphology and/or structure changes; and the intestinal barrier mucosal integrity of the colon. We then observed the regulatory effects of NBD on the composition of the gut microbiota and BA metabolites by using 16S rDNA gene sequencing and targeted metabolomics technology, respectively, and further confirmed their relationship by using systematic Spearman’s correlation analysis. Moreover, we detected the expression of the FXR receptor in colon tissues and the NLRP3 signaling pathway for illuminating the potential mechanism of NBD in alleviating UC. Finally, we used FXR antagonist Z-guggulsterone to confirm the involvement of NBD-medicated FXR activation in DSS-induced UC. This study investigates the protective effects and potential mechanisms of NBD on UC through the gut barrier and provides a theoretical basis for the development of safe and effective novel anti-UC drugs.

## Material and methods

### Reagents

DSS (Catalog # 0216011080) was purchased from MP Biomedicals (Santa Ana, CA, USA); Z-guggulsterone (Catalog # CAS 39025–23-5) was purchased from Yuanye Bio-Technology Co., Ltd (Shanghai, China). Salazosulfapyridine (SASP; Catalog # 09170907) was purchased from Shanghai Xinyi Tianping Pharmaceutical Co. (Shanghai, China); enzyme-linked immunosorbent assay (ELISA) kits for mouse interleukin-6 (IL-6; Catalog # 431304), mouse interleukin-1β (IL-1β; Catalog # 432604), and mouse tumor necrosis factor-α (TNF-α; Catalog # 430904) were purchased from BioLegend Technology Co. (San Diego, CA, USA); antibodies against Caspase-1 (Catalog # 3866S) and cleaved caspase-1 (Catalog # 89332S) were purchased from Cell Signaling Technology (Danvers, MA, USA); antibodies against glyceraldehyde-3-phosphate dehydrogenase (GAPDH; Catalog # GB15004), ZO-1 (Catalog # GB111981), and Occludin (Catalog # GB111401) were purchased from Servicebio Co. (Wuhan, Hubei, China); antibodies against FXR (Catalog # ab155124) and NLRP3 (Catalog # ab263899) were purchased from Abcam (Cambridge, UK).

### Preparation of NBD

NBD consisted of 10 g of Baitouweng (Glycyrrhiza uralensis), Huangbai (Phellodendron chinense C.K. Schneid), Danggui (Angelica sinensis (Oliv.) Diels), Mudanpi (Paeonia suffruticosa Andrews), Muxiang (Aucklandia lappa DC); 15 g of Qinpi (Fraxinus chinensis Roxb), Baishao (Paeonia lactiflora Pall), Chishao (Paeonia veitchii Lynch), Zicao (Lithospermum erythrorhizon Siebold & Zucc), Diyu (Sanguisorba officinalis L), Xianhecao (Agrimonia pilosa Ledeb); 3 g of Huanglian (Coptis chinensis Franch); 5 g of Gancao (Glycyrrhiza uralensis Fisch). All plant names had been checked at https://www.theplantlist.org. All herbs were purchased from the Jiangsu Provincial Hospital of Traditional Chinese Medicine (Nanjing, Jiangsu, China) and accredited by pharmacologist Professor Cao Yuan.

All herbs of NBD were soaked in 10 times the amount of - distilled water for 1 h, decocted for 1 h, and filtered to extract the supernatant. Subsequently, eight times the amount of distilled water was added again and decocted for 30 min. Afterward, the two decoctions were mixed, filtered, and concentrated to 1 g/mL for subsequent use.

### Animals

Male C57BL/6 mice (6 weeks old, 22 ± 2 g) were purchased from Qinglongshan Animal Breeding Farm (Jiangning District, Nanjing, China, license no: SYXK (Su) 2018–0049). Animals were reared at room temperature (22 ± 2°C), humidity 50% ± 5%, and a 12 h/12 h light/dark cycle. All the animal experiments were performed in accordance with the Guide for the Care and Use of Laboratory Animals by the National Institutes of Health. The animal experimental procedures were approved by the Ethics Committee of Nanjing University of Chinese Medicine.

### Establishment of the UC model and treatment

After adaptive feeding for 1 week, all mice were randomly divided into five groups: control group, DSS group, DSS + SASP group, DSS + NBDL (NBD low) group, and DSS + NBDH (NBD high) group. Except for the control group, which was given water, mice in the other four groups were given 3% DSS for 7 days to establish the UC model. Mice in the DSS + NBDL group and DSS + NBDH group received a low dose (12.35 g/kg) or a high dose (24.7 g/kg) of NBD by oral gavage for 10 days. All dose calculations followed the dose-conversion guidelines between humans and experimental animals (the drug-conversion coefficient between adults and mice was 9.1). Based on the estimated adult body weight of 60 kg, the crude drug dosage of high-dose NBD mice was converted to 24.7 g/kg/day and half of the high dose was administered as the low dose. The FXR selective inhibitor Z-guggulsterone was dosed by intraperitoneal injection 100 µL daily at 30 mg/kg. On the 11th day, the mice were euthanized and the blood was taken for serum collection. The colons were removed, rinsed with phosphate-buffered saline (PBS), and measured for length after feces harvest. Then, the part of the colon was separated and stored in 4% paraformaldehyde. The serum, colons, and feces were cryopreserved at −80°C until further analysis.

### Measurement of colon length

The colon length was recognized as being between the inferior end of the ileocecal valve and the end of the colon. The length was measured against a ruler.

### DAI score

During the experiment, the body weight, stool consistency, and presence of occult blood in feces were recorded daily to illustrate the overall status of the mice. The DAI scores were calculated according to the previous report [17]. The scoring system is described in Table 1.

**Table 1.** DAI score

Weight loss (%)	Stool consistency	Rectal bleeding	Score
None	Normal	Occult blood test negative	0
1–5	Mild soft stool	Occult blood test weak positive	1
5–10	Several soft stool	Occult blood test positive	2
10–15	Mild diarrhea	Occult blood test strong positive	3
>15	Severe diarrhea	Gross bleeding	4

### Histological evaluation

Colon tissues (∼1 cm) were immediately fixed in 10% buffered formalin after being rinsed with ice-cold PBS. Paraffin-embedded sections (4 μm) were stained with hematoxylin and eosin staining (H&E) and then examined by using a light microscope to evaluate the colon morphology, mucus layer, and goblet cells. Then, histological scores were calculated by following the scoring criteria in Table 2. A score was assigned to each category based on the observation of H&E staining. The final histological score was calculated as the sum of all scores from each category.

**Table 2.** Evaluation of histological score

Inflammation	Inflammatory extent	Crypt damage	Involvement percentage (%)	Score
None	None	None	None	0
Slight	Mucosa	Basal 1/3 damaged	1–25	1
Moderate	Mucosa and submucosa	Basal 2/3 damaged	26–50	2
Severe	Transmural	Only surface epithelium intact	51–75	3
–	–	Entier crypt and epithelium lost	76–100	4

### ELISA analysis

The expressions of IL-6, IL-1β, and TNF-α in the mouse serum was measured by using ELISA kits and the results were quantified by using a microplate reader.

### Quantitative real‑time polymerase chain reaction

Total RNA was extracted from colon tissues by using an RNA isolator total RNA extraction reagent (Catalog # R401-01, Vazyme BioTech Co., Ltd, Nanjing, China). Next, the extracted RNA was reverse transcribed into complementary DNA (cDNA). Polymerase chain reactions (PCRs) were conducted by using ChamQ SYBR qPCR Master Mix (Catalog # Q311-02) purchased from Vazyme BioTech Co., Ltd. *Gapdh* served as the internal reference when calculating the relative expression of each target gene by using the 2^−ΔΔCt^ method. The primer sequences for the target genes are shown in Table 3.

**Table 3.** Sequences of primers

Gene		Sequences of primers
*Il1b*	F	TGAAATGCCACCTTTTGACAGTGAT
	R	TGATGTGCTGCTGCGAGATTT
*Il6*	F	TAGTCCTTCCTACCCCAATTTC
	R	TTGGTCCTTAGCCACTCCTTC
*Tnf*	F	ATGAGAGGGAGGCCATTTG
	R	CAGCCTCTTCTCATTCCTGC
*Ocln*	F	ACTGGGTCAGGGAATATCCA
	R	TCAGCAGCAGCCATGTACTC
*Tjp1*	F	ACTGGGTCAGGGAATATCCA
	R	TCAGCAGCAGCCATGTACTC
*Gapdh*	F	AGGTCGGTGTGAACGGATTTG
	R	TGTAGACCATGTAGTTGAGGTCA

### Western blot analysis

Colonic tissues were completely homogenized with radio immunoprecipitation assay (RIPA) lysis buffer (Catalog # P0013B, Beyotime Biotech Co., Ltd, Shanghai, China). Next, the extracted proteins were separated by using 10% sodium dodecyl sulfate–polyacrylamide gel electrophoresis and transferred to polyvinylidene fluoride (PVDF) membranes. The membranes were submerged for 2 h in a blocking solution before incubation with the antibodies against GAPDH, FXR, NLRP3, caspase-1, or cleaved caspase-1. Then, after being washed with Tris-buffered saline Tween three times, all PVDF membranes were incubated with secondary antibodies. The bands were exposed to an enhanced chemiluminescence reagent (Catalog # 36222ES76, Yeasen Biotech Co., Ltd, Shanghai, China) and the results were analysed by using Image J software (National Institutes of Health, Bethesda, USA).

### Immunofluorescence analysis

Immunofluorescence (IF) was used to quantify the inflammation of colon tissues and the function of the intestinal barrier. First, tissues were dewaxed and rinsed, and then incubated with various primary antibodies against occludin or ZO-1 at 4°C overnight. All sections were fully rinsed with PBS and incubated with secondary antibodies at room temperature for 1 h. Following PBS rinsing again, the sections were sealed with an immunofluorescence anti-quenching sealing tablet containing 4′,6-diamidino-2-phenylindole (Catalog # C1002, Beyotime Biotechnology, Shanghai, China). Finally, confocal images were observed by using laser scanning confocal microscopy (Leica, Wetzlar, Germany). The results were quantitatively analysed by using ImageJ.

### 16S rDNA sequencing and sequencing data analysis

The DNA extracts of the colon contents were sent to Majorbio Bio-Pharm Technology Co. Ltd (Shanghai, China) for 16S rDNA sequencing. The microbial diversity detection was performed on the V3-V4 hypervariable region of the 16S rDNA of the bacteria. The sequencing was performed with the PE300 sequencing strategy on the Illumina MiSeq platform (Illumina, San Diego, USA).

The SILVA (version 138) database was used to compare the bacterial diversity. Observed taxonomic unit (OTU) clustering of non-repeat sequences was performed by using Uparse software (version 7.0.1090) according to 97% similarity and chimeras were removed. According to the silva138/16s_bacteria species classification database, OTU representative sequences of 97% similar levels were compared with the Ribosomal Database Project (RDP)- classifier Bayesian algorithm. With the UniFrac algorithm, principal coordinate analysis (PCoA) was performed to compare the beta diversity of the species community among samples. Classifications of bacteria among different groups at the phylum and genus levels were compared by using the Kruskal–Wallis rank-sum test. The dominant bacterial communities with statistical differences were analysed by using the line discriminant analysis (LDA) effect size (LEfSe).

### Targeted metabolomics analysis

The standard BA was accurately weighed. Each BA (1 mg) was dissolved in methanol and maintained at a constant volume of 1 mL. After vortex mixing, it was diluted with 50% methanol to obtain BA calibrators of different concentrations. With the mass spectrum peak area of the standard solution as the ordinate, the linear regression standard curve was drawn with the concentration of the standard solution (*r* > 0.99). The mass peak area of the sample analyte was substituted into the linear equation to calculate the concentration result.

The fecal levels of BAs were determined by using the AB SciexEx-ionLC™AD HPLC/SciexQTRAP^®^ 6500 MS system (AB Sciex, Framingham, MA, USA) with an electrospray negative ionization source. Briefly, 50 mg of feces were suspended in 450 μL of ultrapure water. Thereafter, 50 μL of each diluted sample was precipitated with 250 μL of acetonitrile/methanol (v/v = 4:1). The mixture was vortexed for 30 s and centrifuged at 12,000 rpm for 10 min at 4°C. After homogenization and centrifugation, the supernatant was transferred to a sample vial and separated on an AgelaVenusil MPC18 chromatography column (inner diameter of 2.1 × 100 mm, length of 2.5 µm). The samples were eluted by using a gradient of water and acetonitrile with 0.1% formic acid at a flow rate of 0.5 mL/min. BA metabolites were detected by using liquid chromatography–mass spectrometry under the multiple reaction monitoring mode and quantified based on the respective standard curves. MS data were processed by using the SCIEX OS software and analysed with Microsoft Excel. The multivariate data matrix was imported into SIMCA 13.0 (Umetrics, Umeaa, Sweden) for visualization.

### Statistical analysis

All results were displayed as the mean ± standard error of the mean (SEM). Plots were generated by using Prism Graphpad software. Analysis of variance (ANOVA) was used to analyse the differences between the groups. A value of *P *< 0.05 was indicative of statistical significance.

## Results

### NBD ameliorated symptoms and histopathologic changes in DSS-induced UC mice

**Figure 1.**
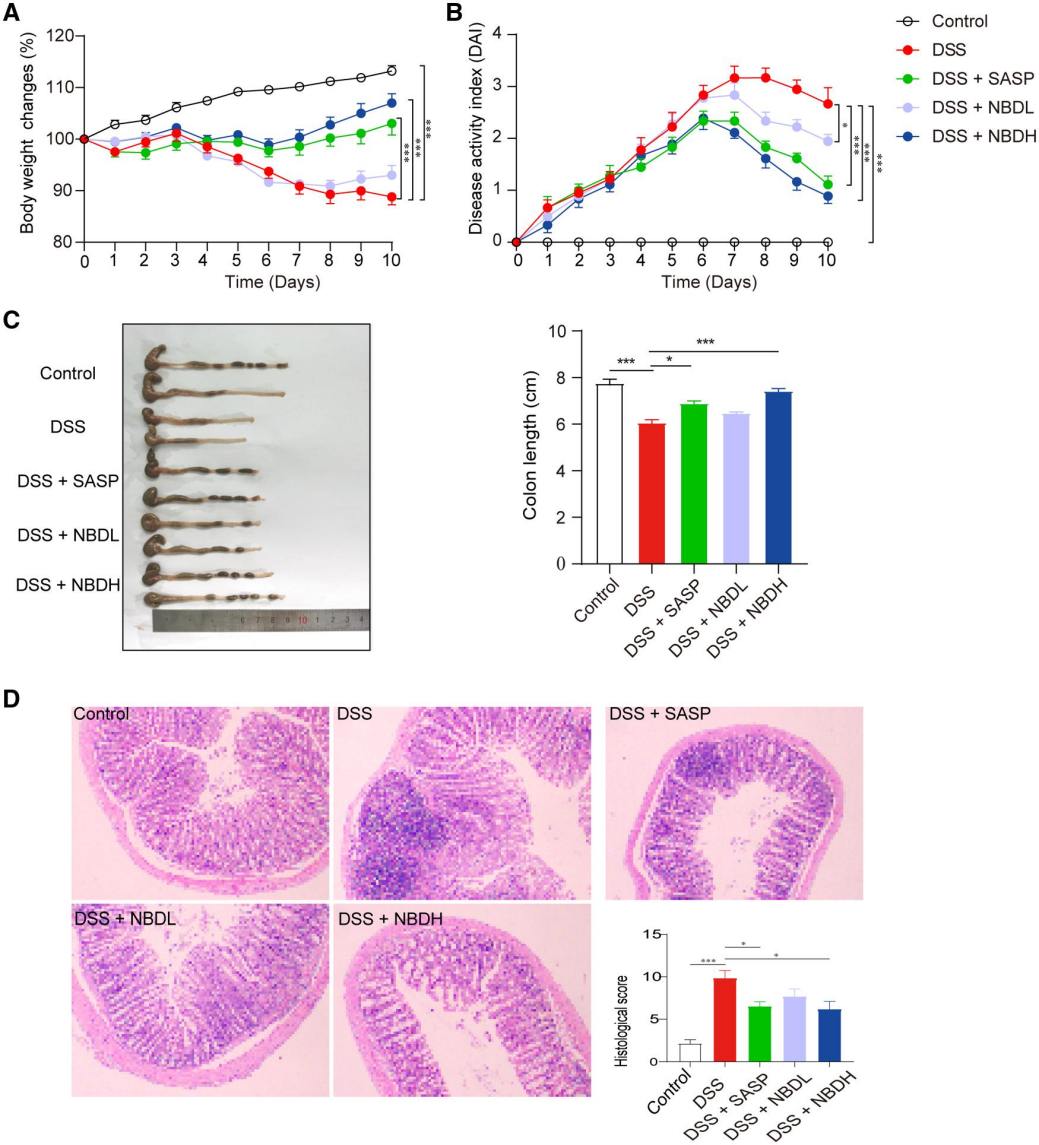
NBD alleviated DSS-induced colitis in mice. (A) Normalized body weight of mice with indicated treatment to corresponding mice on Day 0. (B) DAI score of mice with indicated treatment over 10 days. (C) Representative image of colon morphology and length (left) and bar chart showing the lengths of the colon from various groups (right). (D) Representative images of HE-stained histologic sections (100×) and corresponding pathology scores of various groups. Data are all expressed as means ± SEM (*n *= 6). Statistical analysis was performed by using one-way ANOVA. In (A) and (B), one-way ANOVA was performed by using data on Day 10. *P < 0.05; ***P < 0.001 was considered statistically significant. DSS, SASP, NBDL, and NBDH indicate dextran sodium sulfate, salazosulfapyridine, NBD low dose, and NBD high dose, respectively.

To understand the effect of NBD on DSS-induced UC in mice, we measured the mouse weight, DAI scores, and colon length to indicate the status of the UC. The results showed that mice in the DSS group exhibited significant weight loss, rectal bleeding, shortened colon, and increased DAI scores compared with those in the control group (Figure 1A–C), suggesting that we had established the DSS-induced UC mouse model. Using this model, we further investigated the effects of NBD on DSS-induced UC. Compared with the DSS group, the SASP (drug for treating UC), NBDL, and NBDH groups on Day 10 all showed improved weight loss (Figure 1A), decreased DAI scores (Figure 1B), and increased colon length (Figure 1C), with the NBDH group displaying the best effect.

In addition, the severity of the colonic injury and inflammation were further assessed by using H&E staining. Compared with the control group, the DSS group showed destruction of the mucosal layer, substantial inflammatory cell infiltration, and crypts structural changes (Figure 1D). In the DSS + SASP group, some glands were seen to be missing, a small number of inflammatory cell infiltrations, and a noticeable reduction in pathology scores compared with the DSS group. In the DSS + NBDL group, the pathological condition of the colons was improved compared with those in the DSS group, with a diminished presence of inflammatory cells and neatly arranged glands. In terms of the DSS + NBDH group, the colons were almost restored, the glands were neatly arranged, and no obvious inflammatory cell infiltration was observed. Accordingly, the histopathological scores were significantly increased in the DSS group (9.83 ± 0.91) compared with the control group (2.16 ± 0.48). When treated with NBDH, the scores became significantly lower compared with DSS (6.17 ± 0.95 vs 9.83 ± 0.91). Together, these results suggested that NBD treatment effectively attenuated DSS-induced UC in the mouse.

### NBD attenuated DSS-induced intestinal inflammation

**Figure 2.**
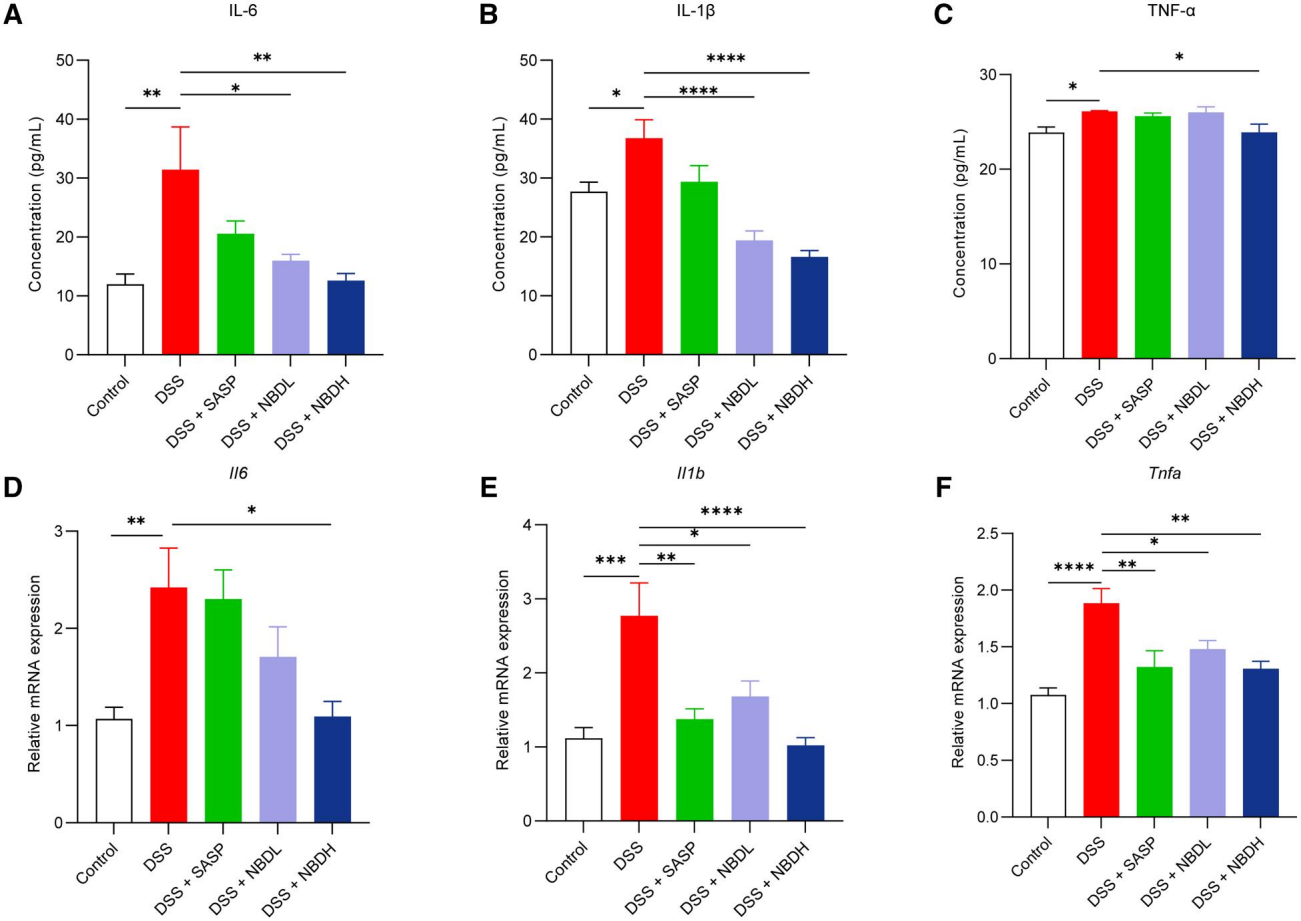
NBD reduced the levels of pro-inflammatory factors in DSS-induced UC mice. (A–C) ELISA for detecting the amount of (A) IL-6, (B) IL-1β, and (C) TNF-α in serum from mice with indicated treatment. (D–F) Quantitative real‑time polymerase chain reaction- for examining the relative mRNA levels of (D) *Il6*, (E) *Il1b*, and (F) *Tnf* in colon tissue from mice with indicated treatment. Data are all expressed as mean ± SEM (*n *= 12). Statistical analysis was performed by using one-way ANOVA. *P < 0.05; **P < 0.01; ***P < 0.001; ****P < 0.0001 was considered statistically significant.

UC is characterized by diffused inflammation that persists throughout the course of the disease. To understand whether NBD improved UC via inhibiting the pro-inflammatory functions of DSS, the amount of pro-inflammatory cytokines IL-6, IL-1β, and TNF-α in serum and colon tissues were measured by ELISA and qPCR, respectively. We observed that the production of IL-6, IL-1β, and TNF-α was significantly elevated in serum from the DSS group compared with those from the control group (Figure 2A–C). The production of IL-6 and IL-1β was largely reduced in the UC mouse upon NBD treatment (Figure 2A and B). However, the enhanced TNF-α was not affected by NBD (Figure 2C). Consistently, the mRNA expression of colon-tissue-derived *Il6*, *Il1b*, and *Tnfa* determined by using qPCR exhibited similar results to the ELISA data: DSS effectively induced the expression of *Il6*, *Il1b*, and *Tnfa*, and such an effect was largely inhibited by NBD treatment (Figure 2D–F). Compared with the inhibitory effect of SASP on the DSS-induced production of pro-inflammatory cytokines, NBDH worked more effectively. In conclusion, NBD could reduce the intestinal inflammatory response caused by DSS.

### NBD repaired intestinal barrier damage in DSS-induced UC mice

**Figure 3.**
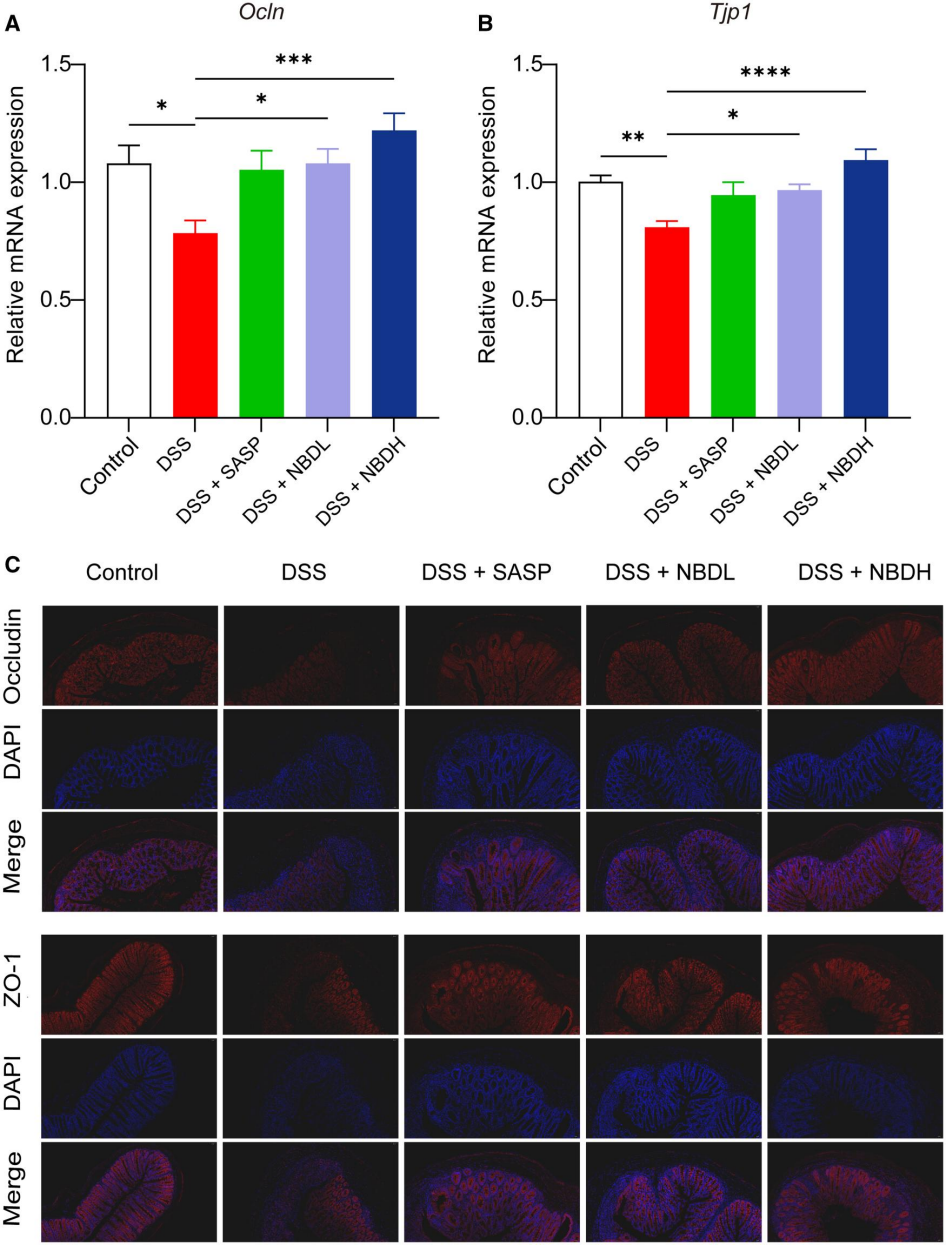
NBD reversed the DSS-mediated reduction of TJ proteins in the colon. (A and B) Relative mRNA expression of (A) *Ocln* and (B) *Tjp1* in colon tissue from mice with indicated treatment was detected by using qPCR. (C) Representative immunofluorescence images of Occludin and ZO-1 staining in colon tissue from mice with indicated treatment. Data in (A) and (B) are expressed as mean ± SEM (*n *= 12). Statistical analysis was performed by using one-way ANOVA. *P < 0.05; **P < 0.01; ***P < 0.001; ****P < 0.0001 were considered statistically significant.

The integrity of the intestinal epithelium was primarily assessed by analysing the colonic expression of tight junction (TJ) proteins, including Occludin and ZO-1. We observed that the mRNA expression levels of *Ocln* (encoding Occludin) and *Tjp1* (encoding ZO-1) in colon tissue was reduced after DSS feeding (Figure 3A and B). However, NBDH treatment significantly inhibited the DSS-induced reduction of both markers (Figure 3A and B).

The above observation was consistently translated into protein staining. As shown by the immunofluorescence in the control group, Occludin and ZO-1 proteins were localized in the membrane of healthy intestinal epithelial cells (Figure 3C). However, most of them were absent in the DSS group, which was indicative of damaged epithelial cells induced by DSS. In contrast, NBD treatment caused substantial re-emergence of these TJ proteins in the mucosal muscularis propria (Figure 3C). These results suggested that NBD could improve intestinal barrier damage and restore intestinal barrier function via regulating the expression of TJ proteins Occludin and ZO-1.

### NBD altered the structure of gut microbiota in UC mice

**Figure 4.**
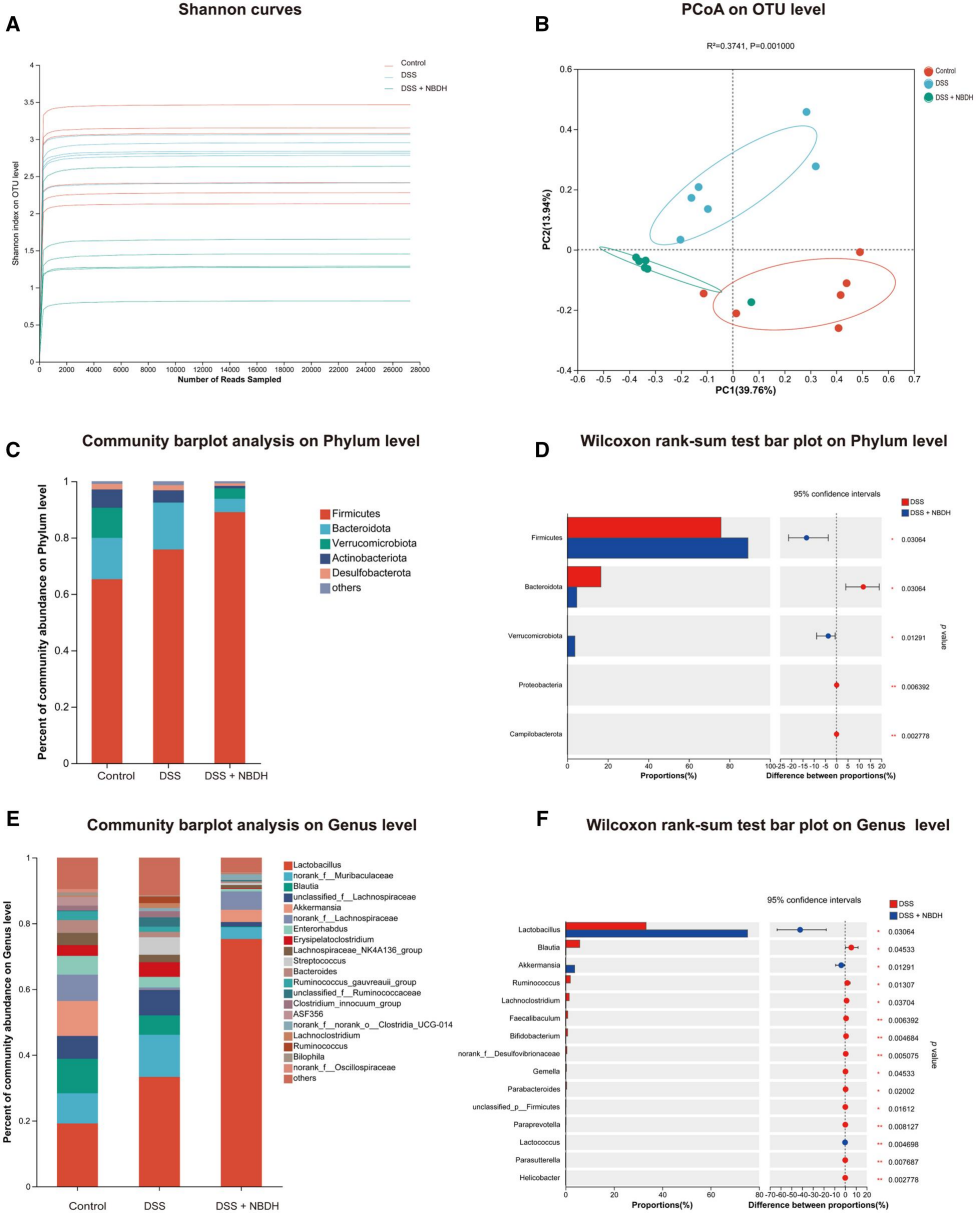
NBD altered the composition of gut microbiota. (A) Shannon index curves and (B) PCoA analysis of each mouse with indicated treatment. (C) Fecal microbiota composition at the phylum level. (D) Phylum-level abundance analysis of fecal microbiota. (E) Fecal microbiota composition at the genus level. (F) Genus-level abundance analysis of fecal microbiota.

To further investigate the effect of NBD on gut microbes, we determined the composition of gut microbes by using 16S rDNA sequencing. The Shannon index curves all increased rapidly and plateaued afterward, confirming that the metagenomic data were able to provide reliable information on bacteria diversity (Figure 4A). PCoA showed that the gut microbiota of DSS-treated mice was clustered significantly differently from that of control mice. The intestinal bacteria of UC mice in the NBD treatment group (DSS + NBDH) were closer to those of the control group, indicating that NBD had altered the composition of the intestinal microbiota affected by DSS (Figure 4B). The compositional proportions of the phylum-level community showed that the dominant bacteria were *Firmicutes* and *Bacteroidota* in the control, DSS, and DSS + NBDH groups. In addition, *Verrucomicrobiota*, *Actinobacteriot*a, and *Desulfobacterota* maintained a sensible proportion in all three groups (Figure 4C). Compared with the DSS group, NBD treatment significantly increased the abundance of *Firmicutes* and *Verrucomicrobiota* and decreased the abundance of *Bacteroidota* and *Proteobacteria* (Figure 4D). The composition ratio of the genus-level community showed that the dominant bacterium was *Lactobacillus* in the control, DSS, and DSS + NBDH groups. In addition, the second dominant bacterium was *Akkermansia* in the control group, unclassified_f_*Lachnospiraceae* in the DSS group, and norank_f_*Lachnospiraceae* in the DSS + NBDH group (Figure 4E). Compared with the DSS group, NBD treatment significantly increased the abundance of *Lactobacillus*, *Akkermansia*, and *Lactococcus*. Meanwhile, the abundance of *Erysipelatoclostridium*, *Blautia*, *Faecalibaculum*, *Lachnoclostridium*, *Bifidobacterium*, and *Norank_f_Desulfovibrionaceae* was significantly decreased (Figure 4F).

**Figure 5.**
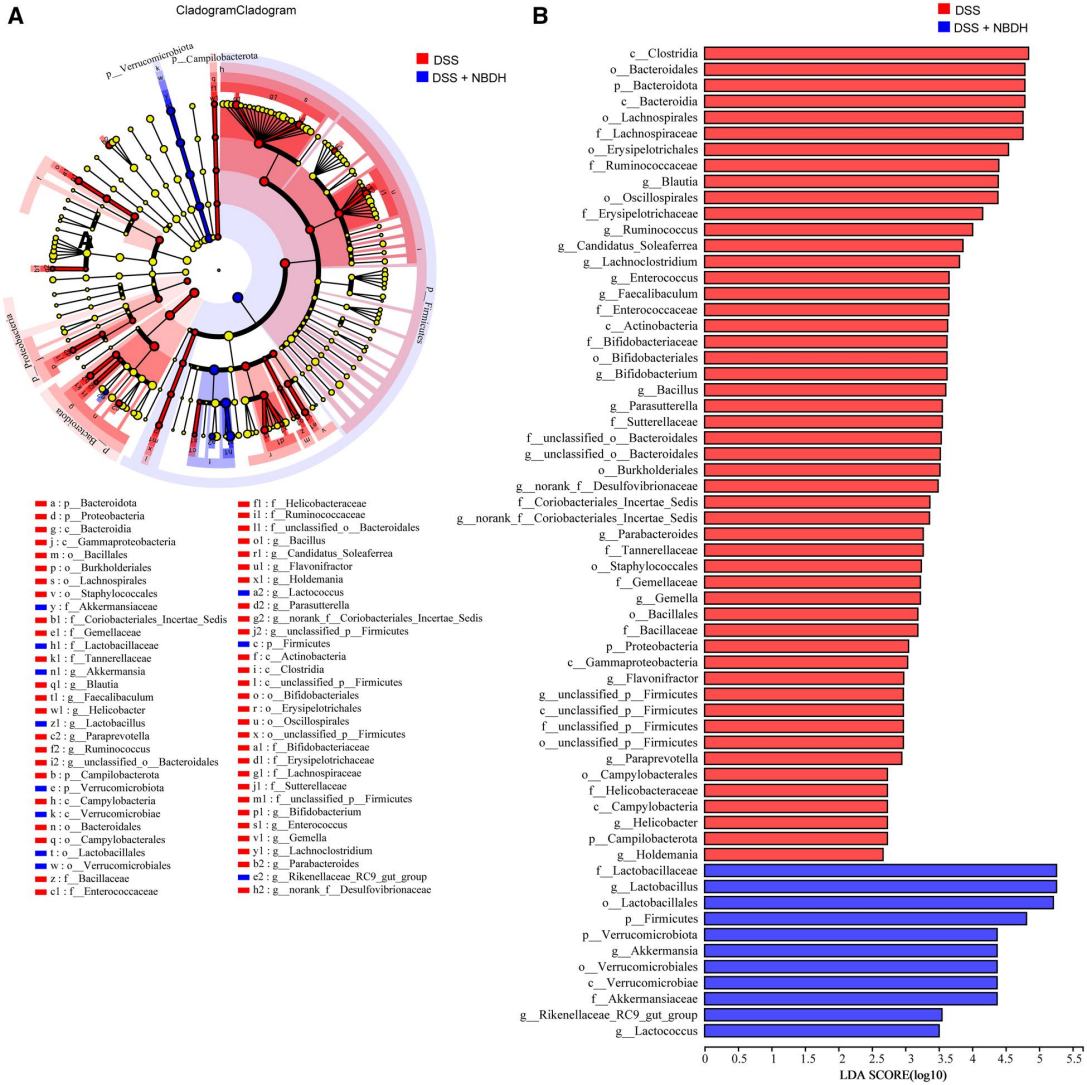
Differences in dominant microorganisms between the DSS and DSS + NBDH groups. (A) LEfSe multilevel species based on LDA score of >2.0. (B) LDA discriminant bar chart (*n *= 6).

In addition, we compared the core bacterial phenotypic differences from phylum to genus between the DSS and NBD groups by using LEfSe analysis. The magnitude of the effect of the abundance of each species on the differential effect was identified by setting the cut-off as an LDA value of >2.0. As shown in Figure 5A, 51 key genera were identified in the DSS-induced UC mouse model and 11 key genera were identified after NBD treatment. After NBD treatment, there was a significant differential effect on the colony abundance of *Firmicutes* and *Verrucomicrobiota* at the phylum level, *Lactobacillaceae* and *Akkermansiaceae* at the family level, and *Lactobacillus* and *Akkermansia* at the genus level (Figure 5B). These results suggest that the efficacy of NBD on UC may depend on its modulation of the gut microbiota.

### The correlation between BAs and gut microbiota

**Figure 6.**
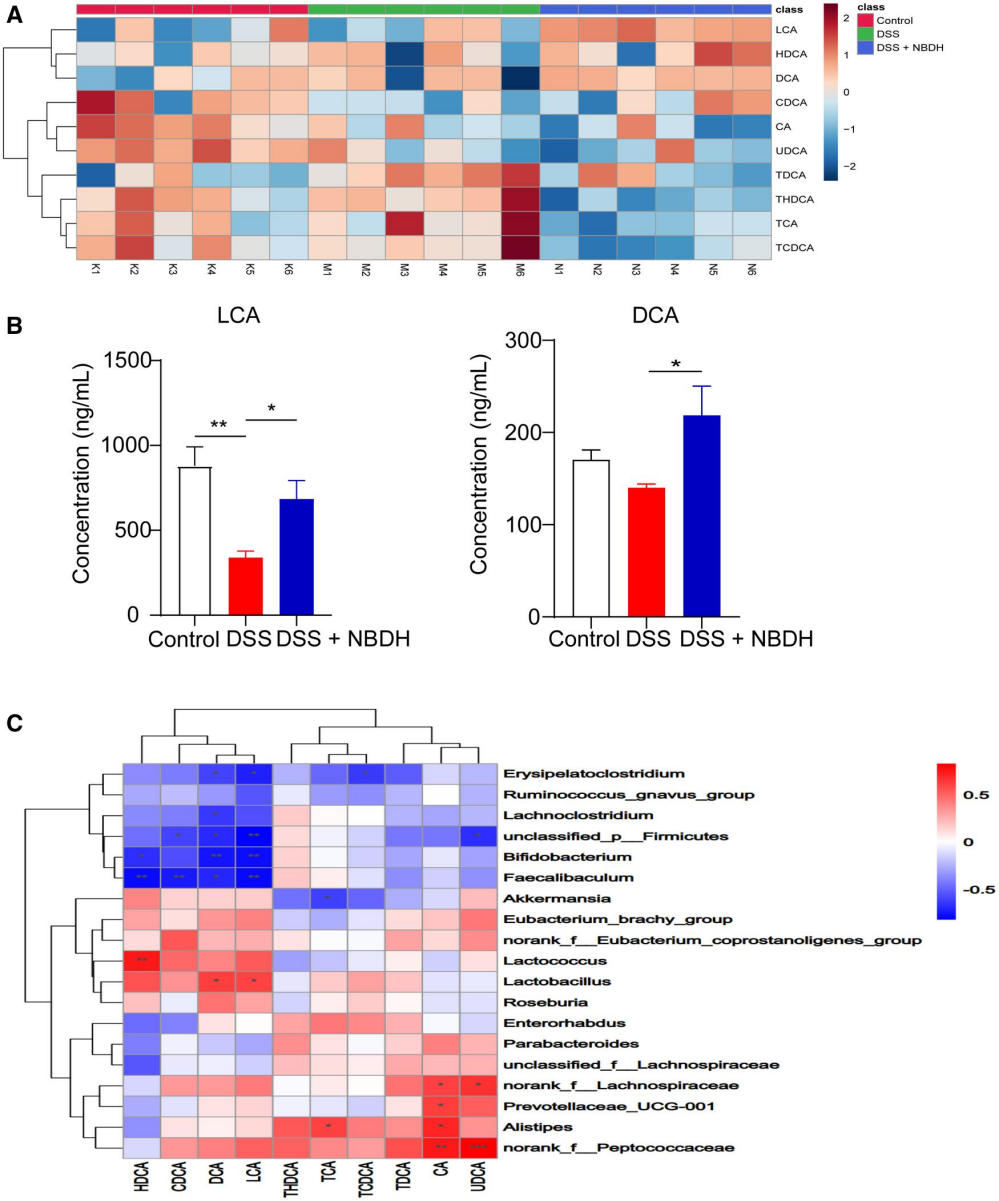
NBD altered BA levels in UC model mice. (A) Heat map of 10 BA levels in mouse feces (*n *= 6). (B) Quantitative analysis of BAs derived from (A). (C) Spearman’s correlation analysis between intestinal flora and BAs. Data are expressed as mean ± SEM (*n *= 6). Statistical analysis was performed by using one-way ANOVA. *P < 0.05; **P < 0.01 were considered statistically significant.

As important players in the host metabolism, BAs not only regulate intestinal homeostasis, but also play a crucial role in the intestinal mucosal barrier to prevent pathogenic antigens. Many studies have shown that gut microbiota ecological dysregulation and BAs disorders impair the intestinal barrier and immunity. To investigate whether NBD treatment affected BA metabolism, we analysed fecal BAs in mice by using the LC–MS/MS method. The BA profiles in the fecal samples of mice in the control and DSS groups were significantly altered, whereas the administration of NBD modulated the DSS-mediated BA metabolism disorders (Figure 6A). Further quantitative analysis of the BAs revealed that DSS-induced reduction of LCA and DCA BAs was significantly enhanced by treatment with NBD (Figure 6B).

To determine whether changes in the fecal BA levels in UC mice were closely associated with gut ecological dysbiosis, we conducted Spearman’s correlation analyses between fecal BAs and intestinal flora genera (Figure 6C). The results showed that the levels of DCA and LCA were positively correlated with the abundance of *Lactobacillus* and negatively correlated with *Erysipelatoclostridium*, *Faecalibaculum*, and *Bifidobacterium*. The above results suggested a close interplay between intestinal flora and fecal BAs.

### NBD ameliorates UC through the FXR–NLRP3 signaling pathway

**Figure 7.**
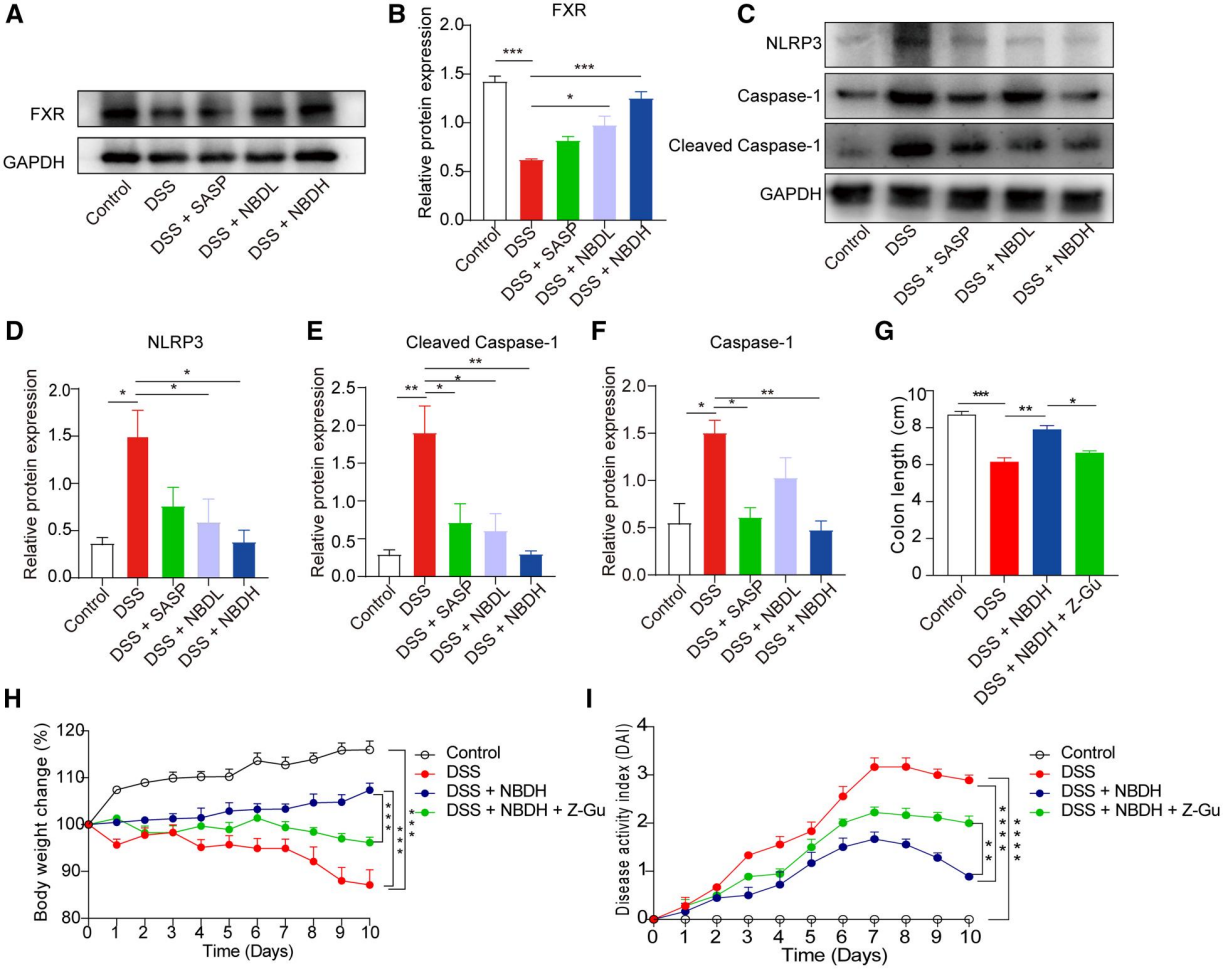
NBD promoted FXR expression but affected the NLRP3 inflammatory pathway in UC mouse model. (A and B) Representative immunoblot and expression analysis of FXR and GAPDH (*n *= 3). (C–F) Representative immunoblot and expression analysis of (D) NLRP3, (E) caspase-1, (F) cleaved caspase-1, and GAPDH (*n *= 3). (G) Bar chart shows the lengths of colons from indicated groups. (H) Normalized body weight of mouse with indicated treatment to corresponding mouse on Day 0. (I) DAI score of mice with indicated treatment over 10 days. Statistical analysis was performed by using one-way ANOVA in (B) and (D–G), two-way ANOVA in (H and I). *P < 0.05; **P < 0.01; ***P < 0.001; ****P < 0.0001 were considered statistically significant.

To understand whether DSS-induced UC was involved in the FXR–NLRP3 pathway, the protein levels of FXR, NLRP3, caspase-1, and cleaved caspase-1 from colon tissues were examined by using Western blot. The protein FXR showed reduced expression in the DSS group compared with the control group (Figure 7A and B). Such an effect was dose-dependently reversed by NBD treatment (Figure 7A and B). In addition, NLRP3, caspase-1, and cleaved caspase-1 showed enhanced expression in the DSS group compared with the control group. The DSS-induced expression of these three inflammatory proteins was significantly reduced after treatment with NBD (Figure 7C–F). We further utilized FXR antagonist Z-guggulsterone to assess whether FXR activation functioned as the mechanism of NBD-medicated UC alleviation. It was observed that, compared with DSS- + NBD, additional Z-guggulsterone treatment resulted in reduced colon length (Figure 7G), lowered body weight (Figure 7H), and worsened DAI scores (Figure 7I). These data suggested that inhibiting FXR could reserve the soothing effect of NBD on DSS-induced UC. Taken together, this suggested that NBD inhibited the NLRP3 inflammasome pathway through the activation of FXR, which is one of the mechanisms for NBD-mediated therapy for UC.

## Discussion

UC is a global challenging disease that is difficult to treat, subject to relapse, and largely affects patients’ life quality [18]. To date, finding new drugs to treat UC is a top priority due to the lack of effective treatments. TCM has the advantages of multiple targets and few side effects; it has saved numerous lives and maintained human health for centuries. Previous clinical studies have confirmed the therapeutic effect of NBD on patients with UC, but the underlying mechanism is still unclear. Therefore, we established the UC model in C57BL/6 mice to explore the mechanism of NBD on UC treatment. For the first time, the effects of NBD on UC were investigated from the perspective of intestinal flora and BA metabolism. The results showed that NBD might control the FXR–NLRP3 signal pathway to alleviate UC by regulating the metabolism of intestinal bacteria and BA.

The pathogenesis of UC is characterized by intestinal mucosal injury and intestinal inflammation. Pro-inflammatory cytokines could aggravate the aggregation and infiltration of neutrophils, macrophages, and other inflammatory cells in colonic tissues. Excessive inflammation causes disruption of the integrity of the intestinal barrier, intestinal edema, and ultimately the formation of ulcers [19]. Thus, restoring intestinal mucosal damage and inhibiting the expression of inflammatory cytokines can effectively alleviate the symptoms of UC. In the study, after drinking 3% DSS water, the mice showed UC-associated symptoms (e.g. colon ulcers, weight loss, severe diarrhea, and hematochezia), which indicated the successful establishment of the UC model. Administration of NBD was found to improve clinical symptoms, colon shortening, and weight loss in UC mice. H&E staining indicated that the UC mice appeared to have colonic injury and intestinal mucosal barrier damage. However, the study demonstrated that NBD could ameliorate intestinal epithelial mucosal damage by decreasing the production of IL-6, IL-1β, and TNF-α inflammatory cytokines, while upregulating the expression levels of Occludin and ZO-1 proteins. Together, NBD repressed inflammation, restored intestinal barrier function, and reduced colon damage in UC mice, suggesting that NBD has potential therapeutic value for UC.

There are 10 trillion bacteria living in the human intestine; the microbiota plays an essential role in human health, body metabolism, and the immune system [20]. As reported in extensive studies, the gut microbiota acts as a crucial player in inflammatory bowel diseases (IBD). The diversity, stability, and clusters of microbiota in IBD patients have been reported to be noticeably varied as compared with healthy controls [21]. In order to examine the effect of NBD on the gut microbiota of UC mice, the 16S rDNA gene sequence was used to analyse the species composition, abundance, and diversity of the microbiota in the feces of mice. Firstly, we analysed the similarities and differences in gut microbiota composition by using PCoA and the results showed that the DSS-induced intestinal flora composition of UC mice was significantly changed, but the gut microbiota of mice in the NBD group tended to a certain extent towards that in the control group, indicating that NBD could restore the intestinal microflora composition of UC mice and regulate the homeostasis of intestinal flora. Then, further analysis of the gut bacteria at different classification levels found that DSS destroyed the abundance and altered diversity of intestinal flora. Compared with the control group, DSS treatment led to shifts in gut microbiota composition, with a decrease in *Bacteroidetes* and an increase in *Proteobacteria*, which was consistent with previous reports [4]. LDA results showed that, in the DSS group, *Proteobacteria*, *Erysipelotrichaceae*, and *Enterococcus* might be the cause of colonic inflammation. *Proteobacteria* are believed to be the main pathogenic bacteria in intestinal microbes and their increased abundance may lead to microbial imbalance and increase the risk of disease [22, 23]. Studies have shown that the abundance of *Erysipelotrichaceae* in the intestinal flora of UC mice in the acute stage has increased significantly, suggesting that *Erysipelotrichaceae* is associated with inflammation and may promote the development of UC [24]. In a previous study, the abundance of *Enterococcus* in UC patients was significantly higher than that of normal subjects, multiple strains isolated from UC patients were sequenced, and it was speculated that *Enterococcus faecium* might be a bacterial type that causes inflammation [25]. Compared with the DSS group, we found that the NBD treatment could increase the abundance of *Firmicutes*, *Lactobacillus*, and *Akkermansia*, which displayed a negative correlation with pro-inflammatory factors. Meanwhile, NBD decreased the abundance of pathogenic bacteria, including *Proteobacteria*, *Erysipelotrichaceae*, and *Enterococcus*, which showed a positive correlation with pro-inflammatory factors. *Lactobacillus* is a probiotic and one study showed that *Lactobacillus* improved colitis in mice by increasing the thickness of the colon mucus [26]. *Akkermansia* has been shown to protect mice from DSS-induced colitis by maintaining the intestinal barrier and down-regulating inflammatory cytokines and chemokines through microbe–host interactions or by improving the microbial community to improve mucosal inflammation [27]. In conclusion, NBD regulates the abundance and diversity of gut microbiota by increasing the level of beneficial bacteria and decreasing the level of pathogenic bacteria to regulate intestinal homeostasis, which alleviates DSS-induced UC.

The pathogenic role of BAs in UC has been widely recognized. Dysregulation of BA metabolism causes the disruption of intestinal homeostasis, such as immunity imbalance and dysbacteriosis, leading to gut damage [28]. Intestinal BA metabolism is significantly regulated by the gut bacteria. As potent signaling molecules, BAs have a complex interaction with the intestinal microbiota [20]. On the one hand, the gut microbiota was involved in the synthesis, biotransformation, and metabolism of BAs. On the other, BAs regulate the composition of the microbiota in indirect or direct ways, such as through the protective effect of intestinal barriers. It is the first time that we had explored the effects of NBD on gut microbiota composition and BA metabolites in a UC model. In our study, NBD played a unique role in maintaining the stability of the gut microbiota and regulating BA metabolism. NBD treatment enriched the diversity and composition of the intestinal microbiota and increased the abundance of beneficial bacteria. By comparing the levels of BAs in the stools of mice, it was found that DSS-induced UC destroyed the content of BAs in the stools, mainly decreased the levels of DCA and chenodeoxycholic acid (CDCA) and increased the levels of binding BAs taurodeoxycholic acid, taurohyodeoxycholic acid, taurocholic acid, and taurochenodeoxycholic acid. We observed that NBD increased LCA, DCA, and chenodeoxycholic acid (CDA) levels, which were related to the regulation of intestinal microbiota. According to Spearman’s correlation analysis, the levels of DCA and LCA had a favorable correlation with the abundance of *Lactobacillus* and *Akkermansia*. Furthermore, in the DSS group, the relative abundance of *Erysipelatoclostridium* was negatively linked with several levels of BA, indicating that it might encourage abnormalities of BA metabolism. *Lactobacillus* and *Akkermansia* are the two core bacterial phenotypes in the NBD group and both showed the ability to regulate BA metabolism. *Lactobacillus* mediates bile brine hydrolysase, which is involved in BA dissociation [29]. *Akkermansia* is considered to be a promising probiotic that has been shown to play a critical role in maintaining the integrity of the intestinal mucus layer [30]. *Akkermansia* may upregulate BA-coenzyme production to increase the synthesis of conjugated primary BAs [31]. The decrease in *Erysipelatoclostridium* may contribute to the decreased microbial metabolism of BAs in radiation-induced intestinal injury [32]. In this study, the relative abundance of *Lactobacillus* and *Akkermansia* was positively correlated with DCA and LCA. Furthermore, DCA and LCA, the natural agonists of FXR, have been used to provide therapeutic effects against IBD by restoring intestinal barrier function and reducing inflammation [33]. These results suggest that NBD can regulate DCA and LCA metabolism and reduce inflammation by increasing the abundance of *Lactobacillus* and *Akkermansia*. Therefore, we speculate that NBD may restore the disturbance of BAs mainly by regulating intestinal flora, reducing bound BAs, and promoting free BAs, thus alleviating UC.

To perform molecular signaling, BAs bind to various BA receptors. FXR is a nuclear receptor that can be activated by BAs. LCA and DCA are the main ligands of FXR. FXR acts as a key transcription factor for the regulation of the BA enterohepatic circulation, thereby maintaining BA homeostasis by controlling BA synthesis, influx, and efflux [34]. FXR activity is also regulated by the flora and some intestinal flora can promote FXR signaling in mice by unbinding the FXR antagonist T-β-MCA [35]. FXR also has a regulatory effect on the composition of intestinal flora, mainly by indirectly inhibiting the overgrowth of bacteria and mucosal damage in the ileum through the regulation of BAs. Studies have shown that the downregulation of FXR leads to a significant increase in the abundance of *Bacteroides fragilis* enterotoxin, causing cancer-promoting and multistage inflammatory responses [36]. The expression of FXR decreased with the development of UC and UC-associated cancer, and its low expression could also be relieved with the remission of UC [10]. We investigated the effect of NBD on FXR activation in colon tissues. As expected, the protein expression level of FXR was significantly increased in NBD-treated mice. These results suggest that NBD alleviates inflammation via improvement in the gut microbiota and BA dysmetabolism, and its anti-inflammatory effect may be through activating colon FXR activity.

Studies have shown that FXR activation decreases the expression of pro-inflammatory cytokines by inhibiting NF-κB activity and NLRP3 inflammasome assembly [37]. The NLRP3 inflammatory vesicles are ubiquitous in epithelial and immune cells, and are rapidly becoming important regulators of homeostasis in the gut [38]. The formation of the inflammasome complex is mediated by this innate immune receptor, including NLRP3, activating signal cointegrator-2 (ASC-2), and caspase-1, triggering the activation of caspase-1 and secretion of IL-1β and IL-18 in the presence of microbial ligands, and is implicated in the pathogenesis of UC [39]. Hao *et al* [40] found that FXR gene knockout mice were more susceptible to lipopolysaccharide (LPS)-induced NLRP3 inflammation-associated sepsis, while FXR overexpression mice were more resistant to it, indicating that BA activation of the nuclear receptor FXR had an inhibitory effect on NLRP3 inflammation-related sepsis. Further studies have shown that CDCA or the agonist GW4064 can inhibit the activation of endoplasmic reticulum (ER) stress-mediated protein kinase RNA-like ER kinase by activating FXR, and thereby inhibiting the expression and activation of the CCAT enhancer-binding protein homologous protein (CHOP)-dependent NLRP3 inflammasome [41]. These studies suggest that FXR can be used as a potential therapeutic target for NLRP3 inflammation-related diseases, alleviating related intestinal diseases. Recent studies have shown that NLRP3 inflammatory vesicles not only are key elements of host defense, but also regulate intestinal homeostasis by controlling intestinal epithelial integrity and modulating immune responses to the gut microbiota [42]. With the development of inflammation, the expression of the TJ protein (ZO-1 and occludin-1) gene in colon tissue significantly decreases.

In the present study, we found that hyperactivation of intestinal NLRP3 exacerbated experimental UC in mice. NBD downregulated NLRP3, ASC, and caspase-1, and reduced IL-1β secretion in colon tissue; NBD could also reverse the change in the TJ protein. We further observed that the introduction of FXR antagonist Z-guggulsterone could impede the protective roles of NBD in DSS-induced UC.

Therefore, we can preliminarily propose the potential therapeutic mechanism of NBD to treat UC in mice as follows. First, NBD reinstated the microbial imbalance and led to an increase in the abundance of *Lactobacillus* and *Akkermansia* in UC mice. Moreover, it regulated the microbial BA metabolism to increase CDA and LCA levels, which further induced the activation of FXR to greatly improve gut-barrier integrity and inhibit the inflammatory process in mice with UC. Of note, however, blocking FXR activation only displayed a partial inhibition against the protective effect of NBD, hinting that there would be other unknown mechanisms in addition to the FXR–NLRP3 axis regulated by NBD to fight against DSS-induced UC. A possible mechanism has been elucidated in a previous rat study that NBD in combination with fecal microbiota transplantation alleviated DSS-induced UC by regulating NF-κΒ/STAT3 pathways and restoring the intestinal flora [16]. More excitingly, our previous clinical study revealed that NBD, when combined with Western medicines, played a further significantly protective function in patients with UC [15]. All these findings prompted the potential of clinically introducing NBD into the clinical treatment of UC patients.

In summary, the present study confirmed that NBD alleviated DSS-induced UC by regulating the intestinal flora and BAs, and modulating the intestinal mucosal barrier FXR–NLRP3 signaling pathway. Specifically, NBD improved the abundance of intestinal flora in UC mice, regulated the intestinal BA metabolic balance, activated colon FXR, inhibited the NLRP3 inflammasome, downregulated pro-inflammatory factors, restored intestinal barrier function, and improved UC. However, the study has some limitations, including whether the beneficial effects of NBD are expressed primarily by targeting FXR signaling pathways, which still requires more experimental evidence to validate. Secondly, in future studies, we need to determine the active component in NBD that enables the treatment of UC so as to clarify the molecular mechanism of NBD and establish its clinical application. The study provided a new idea for the treatment of UC with NBD, which may be beneficial for the treatment of UC patients in the future.

## Supplementary Material

goaf055_Supplementary_Data

## Data Availability

The experimental data used to support the findings of this study are available from the corresponding author upon request.
